# Differential DNA methylation profiles in gynecological cancers and correlation with clinico-pathological data

**DOI:** 10.1186/1471-2407-6-212

**Published:** 2006-08-23

**Authors:** Hui-Juan Yang, Vincent WS Liu, Yue Wang, Percy CK Tsang, Hextan YS Ngan

**Affiliations:** 1 Department of Gynecologic Oncology, Fudan University Cancer Hospital, Shanghai, China; 2 Department of Obstetrics & Gynecology, University of Hong Kong, Hong Kong SAR, China; 3 Department of Obstetrics & Gynecology, People's Hospital, Peking University, China

## Abstract

**Background:**

Epigenetic gene silencing is one of the major causes of carcinogenesis. Its widespread occurrence in cancer genome could inactivate many cellular pathways including DNA repair, cell cycle control, apoptosis, cell adherence, and detoxification. The abnormal promoter methylation might be a potential molecular marker for cancer management.

**Methods:**

For rapid identification of potential targets for aberrant methylation in gynecological cancers, methylation status of the CpG islands of 34 genes was determined using pooled DNA approach and methylation-specific PCR. Pooled DNA mixture from each cancer type (50 cervical cancers, 50 endometrial cancers and 50 ovarian cancers) was made to form three test samples. The corresponding normal DNA from the patients of each cancer type was also pooled to form the other three control samples. Methylated alleles detected in tumors, but not in normal controls, were indicative of aberrant methylation in tumors. Having identified potential markers, frequencies of methylation were further analyzed in individual samples. Markers identified are used to correlate with clinico-pathological data of tumors using χ^2 ^or Fisher's exact test.

**Results:**

*APC *and *p16 *were hypermethylated across the three cancers. *MINT31 *and *PTEN *were hypermethylated in cervical and ovarian cancers. Specific methylation was found in cervical cancer (including *CDH1, DAPK*, *MGMT *and *MINT2*), endometrial cancer (*CASP8*, *CDH13, hMLH1 *and *p73*), and ovarian cancer (*BRCA1*, *p14*, *p15, RIZ1 and TMS1*). The frequencies of occurrence of hypermethylation in 4 candidate genes in individual samples of each cancer type (*DAPK*, *MGMT*, *p16 *and *PTEN *in 127 cervical cancers; *APC*, *CDH13*, *hMLH1 *and *p16 *in 60 endometrial cancers; and *BRCA1*, *p14*, *p16 *and *PTEN *in 49 ovarian cancers) were examined for further confirmation. Incidence varied among different genes and in different cancer types ranging from the lowest 8.2% (*PTEN *in ovarian cancer) to the highest 56.7% (*DAPK *in cervical cancer). Aberrant methylation for some genes (*BRCA1*, *DAPK*, *hMLH1*, *MGMT*, *p14*, *p16*, and *PTEN*) was also associated with clinico-pathological data.

**Conclusion:**

Thus, differential methylation profiles occur in the three types of gynecologic cancer. Detection of methylation for critical loci is potentially useful as epigenetic markers in tumor classification. More studies using a much larger sample size are needed to define the potential role of DNA methylation as marker for cancer management.

## Background

Hypermethylation of the CpG islands of gene promoter is one of the earliest and most frequent alterations leading to cancer [1,2]. It is an important epigenetic mechanism for gene silencing, which may confer tumor cells of growth advantage [1,3]. Many cellular pathways are inactivated by this epigenetic event, including DNA repair, cell cycle, apoptosis, cell adherence, and detoxification [4]. Evidence showed that CpG island hypermethylation is widespread in human genome. But it is not randomly distributed in carcinogenesis, and is gene-specific and cancer-type specific [5-7]. The specific patterns of CpG island hypermethylation between tumor types may provide a useful signature for tumor diagnosis and prognosis [4,7-9]. Moreover, genes that are frequently aberrantly methylated in specific tumors have been used as molecular targets for the detection of neoplastic cells in body fluids such as urine and plasma which provide additional targets for noninvasive early diagnosis and monitoring for cancers [9,10].

CpG island hypermethylation has been demonstrated in gynecologic cancers [11-16]. However, studies were separately performed in each of the three cancers. Since different genes and different methods were employed, the biological and clinical roles of this epigenetic event in gynecologic cancers were not comparable among studies. However, it is meaningful to compare the role for CpG island hypermethylation in the development and progression of the three cancers because of the correlation in the embryonic development of ovarian epithelium, endometrium and cervix. Moreover, the numbers of CpG islands investigated in those studies were very limited. Recently, microarray-based techniques provide a powerful tool to map methylation patterns in multiple genes and multiple CpG sites within genes [17,18]. However, those advanced techniques are not presently widely available.

For rapid identification of potential targets for aberrant methylation in gynecological cancers as markers for cancer management, thus, in the present study with the technologies at hand, the pooled DNA approach and methylation-specific PCR (MSP) were employed to determine the methylation status of 34 gene loci among the three gynecologic cancers. Markers identified are used to correlate with clinico-pathological data of tumors.

## Methods

### Patients

The study was approved by the Institutional Review Board. Patients of 127 cases of cervical cancer, 60 cases of endometrial cancer and 50 cases of ovarian cancer were recruited for this study. Those patients were diagnosed and treated in Queen Mary Hospital, Hong Kong from January, 1990 to June, 2003. Primary tumor tissues and paired normal tissues were obtained from biopsy or surgical specimens. The paired normal tissues taken from cervical cancer patients are usually blood. From endometrial cancer patients, the normal tissues are mainly cervix. Patients with ovarian cancer, the normal tissues taken are either from the cervix or the endometrium. Finally, the tissues were maintained frozen in our tissue bank. Informed consent was obtained from all individuals for the collection of tissue or blood.

The histological types and grades of tumor were classified according to WHO criteria. The stage of each cancer was established according to the International Federation of Gynecology and Obstetrics (FIGO) criteria. Table 1 lists the clinical characteristics and treatment modality for those patients. All the patients were followed up after the last treatment every 3 months for the first 2 years, every 6 months for the next 3 years, then yearly thereafter. The end of the observation period was August, 2003. Response to treatment was evaluated by physical examination, appropriate imaging studies or biopsy. Persistent disease was defined as detection of disease within 6 months after completion of treatment. Recurrent disease was defined as local (within pelvis) or distant (outside the pelvis) lesions appearing after a disease-free interval of more than 6 months after completion of treatment.

### DNA extraction and preparation of pooled DNA

Genomic DNA from tumor and paired normal tissues was extracted using the standard Proteinase K treatment followed by phenol/chloroform extraction. One μg of tumor DNA from each of the 50 patients with same cancer type (for cervical and endometrial cancer, these 50 patients were randomly selected from the total 127 and 60 patients, respectively) was mixed together as the test DNA pool (M2, M4 and M6 for cervical, endometrial and ovarian cancers, respectively). Similarly, the paired normal DNA from those patients was also pooled to form the controls (M1, M3 and M5 for cervical, endometrial and ovarian cancers, respectively).

### DNA methylation analysis

CpG island methylation status for a total of 34 loci in the three cancers was first assessed in the differential DNA pools representing tumor and normal DNA by MSP. The methylation status of each locus has already been reported in one or multiple cancer types (see Additional file 1). A pair of primers for each locus, which only recognizes methylated sequence, was used to amplify the sequence of interest. Methylated alleles detected in tumor DNA pool, but not in normal DNA pool, were indicative of aberrant methylation in tumors. Furthermore, nine loci, which were aberrantly methylated in tumors suggestive by the above analysis, were selected for subsequent methylation analysis in individual samples including 127 cervical cancers, 60 endometrial cancers and 49 ovarian cancers (DNA was not sufficient in one case). Another set of primer pair for each locus, which only recognizes unmethylated alleles, was added to discriminate the methylated and unmethylated sequences for each sample. A placenta DNA sample treated in vitro with *SssI *methyltransferase (New England Biolabs Inc., Beverly, MA) followed by bisulfite treatment was used as a positive control for methylated allele and as a negative control for the unmethylated allele. A placenta DNA without pre-treatment of *SssI *methyltransferase prior modification, and a placenta DNA without any treatment were used as negative controls for the methylated allele.

### Sodium-bisulfite treatment, MSP and sequencing

The bisulfite treatment of DNA, MSP conditions and sequencing of PCR products have been described in our previous report [16]. Primer sequences and annealing temperature were same as those published reports. Details of the primers, annealing temperature, product size are listed [see Additional file 1].

### Data analysis and statistics

The association between aberrant hypermethylation and clinico-pathological parameters was tested by the χ^2 ^test or Fisher's exact test. The *t*-test was used to compare the mean age of different groups. All variables (age, stage, grade, histological type, and CpG island hypermethylation) were also subjected to multivariate analysis using Logistic regression. Disease-free survival (DFS) was defined as the period from the completion of the last treatment to the date of the first documented evidence of recurrent disease. Patients without recurrent disease were censored at their last follow-up visit or death. Overall survival (OS) was calculated from the date of diagnosis to death; patients who survived until the end of the observation period were censored at their last follow-up visit. Patients who died because of other causes than cancer were censored at their date of death. Survival analysis was performed by the method of Kaplan-Meier, and differences between groups were determined by log-rank test. The Cox proportional hazards model was used for multivariate survival analysis. CpG island hypermethylation, patients' age, FIGO stage, tumor grading and histological type were included in the regression model. All the statistical analysis was performed using the software SPSS for Windows version 13.0. *P *< 0.05 was taken as statistically significant.

## Results

### Identification of CpG island methylation profiles in gynecologic cancers

**Figure 1:**
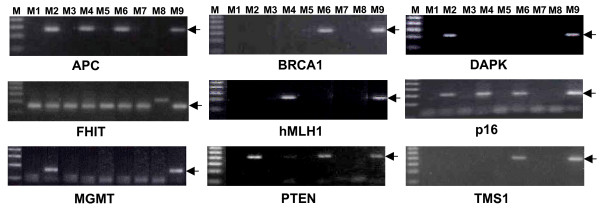
**Identification of CpG island hypermethylation by pooled DNA approach and MSP**. Methylated alleles for some gene loci produced by MSP in tumor and normal DNA pools of the three types of gynecologic cancer. M: 50 bp DNA marker; M1 and M2: normal DNA and tumor DNA pools of cervical cancer; M3 and M4: normal DNA and tumor DNA pools of endometrial cancer; M5 and M6: normal DNA and tumor DNA pools of ovarian cancer; M7: a sodium bisulfite-treated placenta DNA, but without pre-treatment of *Sss*I methyltransferase; M8: an un-treated placenta DNA; M9: a placenta DNA treated with *Sss*I methyltransferase followed by sodium-bisulfite. The arrows indicate the positions of the PCR products.

**Figure 2:**
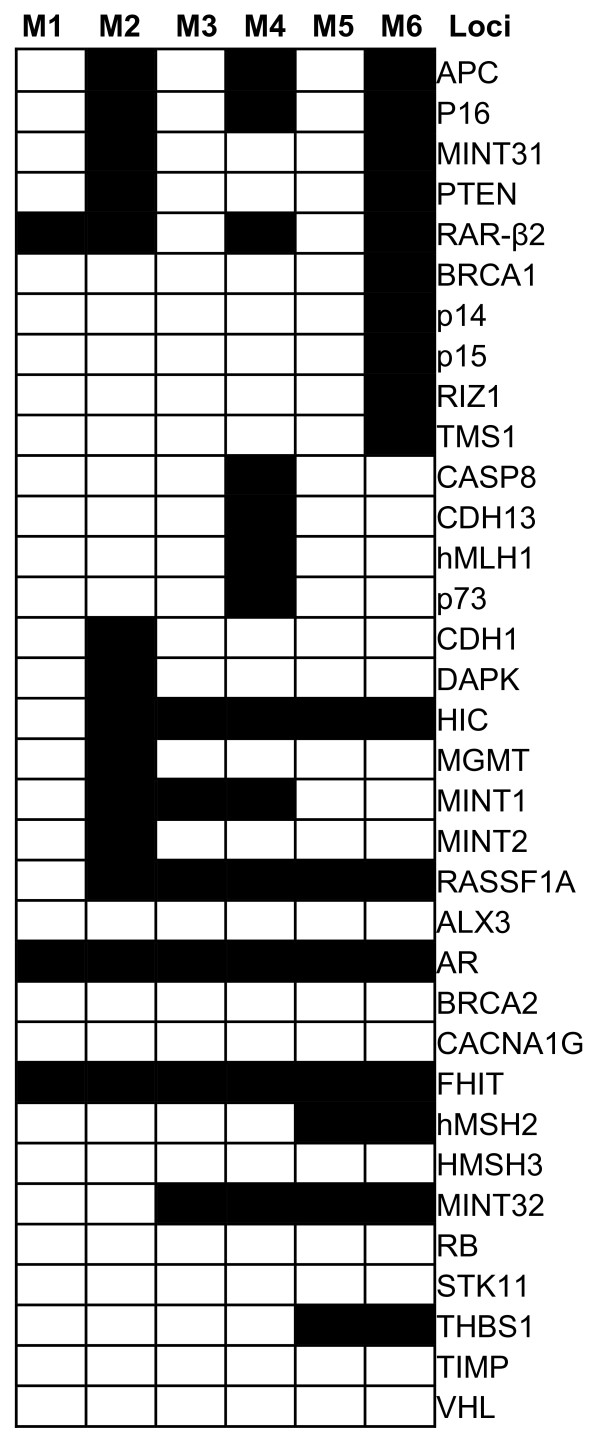
**Methylation status of the 34 gene loci in the three types of gynecologic cancer**. (■) Presence of methylated alleles. (□) Absence of methylated alleles.

Figure 1 shows the methylated alleles for some gene loci produced by MSP in the tumor and normal DNA pools of the three cancers. The methylation status for the 34 loci in the three cancers was summarized in Figure 2. Among those 34 loci, (1) aberrant methylation of *APC *and *p16 *was detected across the three cancers; (2) *MINT31 *and *PTEN *were aberrantly methylated in both cervical cancer and ovarian cancer; (3) specifically, aberrant methylation for *CDH1*, *DAPK*, *MGMT *and *MINT2 *was only present in cervical cancer; *CASP8*, *CDH13*, *hMLH1 *and *p73 *were just methylated in endometrial cancer; aberrant methylation for *BRCA1*, *p14*, *p15*, *RIZ *and *TMS1 *was only detected in ovarian cancer. For the other loci, aberrant methylation was not indicated in any of the three cancers because methylated alleles were present in both tumor and normal DNA (i.e. methylation occurred in that particular gene in both tumor and normal tissues) e.g. FHIT gene (Figure 2), or methylated allele was not detected in both (i.e. methylation did not occur in that particular gene in both tumor and normal tissues) e.g. ALX3 gene (Figure 2).

### Confirmation of hypermethylation for candidate genes in primary tumors

**Figure 3:**
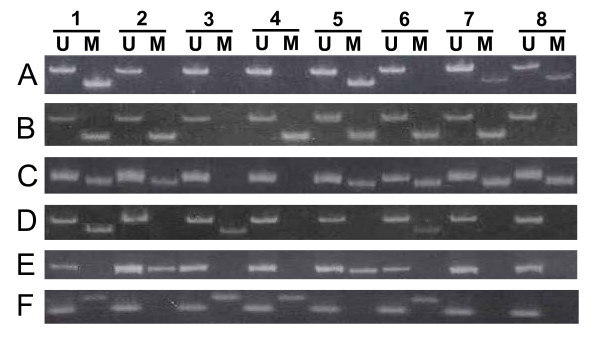
**Methylated (M) and unmethylated (U) alleles for six genes produced by MSP in representative samples**. (**A**) APC: methylated alleles were detected in cases A1, A5, A7 and A8; (**B**) BRCA1: methylated alleles were detected in cases B1, B2, B4, B5, B6 and B7; (**C**) DAPK: methylated alleles were detected in cases C1, C2, C5, C6, C7 and C8; (**D**) hMLH1: methylated alleles were detected in case D1, D3 and D6; (**E**) p16: methylated alleles were detected in cases E2 and E5; (**F**) PTEN: methylated alleles were detected in cases F1, F3, F4 and F6.

For further confirmation, 9 loci with aberrant methylation in the three cancers were further studied by MSP in individual primary tumors. The incidence of methylation for these loci in the three cancers and their association with clinico-pathological parameters and prognosis were also determined. Four loci including *DAPK*, *MGMT*, *p16 *and *PTEN *were selected for methylation analysis in 127 cervical cancers; *APC*, *CDH13*, *p16 *and *hMLH1 *were examined in 60 endometrial cancers; and *BRCA1*, *p14*, *p16 *and *PTEN *in 49 ovarian cancers. Figure 3 shows the methylated and unmethylated alleles produced by MSP in representative samples. Methylation for at least one gene was detectable in 72.4% (92/127) of cervical cancers (Table 2), 66.7% (40/60) of endometrial cancers (Table 3) and 51.0% (25/49) of ovarian cancers (Table 4). Incidence of hypermethylation for *DAPK*, *MGMT*, *p16 *and *PTEN *in cervical cancer was 56.7%, 25.2%, 16.5% and 15.7% respectively. Incidence for *APC, CDH13*, *p16 *and *hMLH1 *in endometrial cancer was 41.7%, 35.0%, 25.0% and 13.3% respectively. Incidence for *BRCA1, p14*, *p16 *and *PTEN *in ovarian cancer was 24.5%, 18.4%, 16.3% and 8.2% respectively. Although we found genes specific for each type of the three tumors, we deliberately chose *p16 *and *PTEN *to determine if the frequencies of methylation are different between different tumors. From the above results, no obvious difference was observed in the methylation frequencies of *p16 *and *PTEN *in the different tumors.

### CpG island hypermethylation in relation to clinico-pathological parameters

Tables 2, 3 &4 show the association between CpG island hypermethylation of the nine loci and the clinico-pathological parameters in the three cancers. Hypermethylation for some specific loci was related to particular tumor subtypes. In cervical cancer (Table 2), *DAPK *gene hypermethylation was more frequently detected in squamous cell carcinoma (SCC) than in adenocarcinoma (AC) (63.3% versus 34.5%, *P *= 0.006), whereas, *PTEN *was more frequently detected in AC than in SCC (34.5% versus 10.2%, *P *= 0.002). The two findings were confirmed by multivariate analysis (*P *= 0.004, and 0.002, respectively). A significant correlation was observed between *MGMT *hypermethylation and the early-stage tumor (*P *= 0.03), but was not confirmed by multivariate analysis (*P *= 0.733).

In endometrial cancer (Table 3), *hMLH1 *gene hypermethylation was more common in moderately or poorly differentiated tumors (G2 & G3) than in well-differentiated tumors (G1) (28% versus 2.8%, *P *= 0.007), which was confirmed by multivariate analysis (*P *= 0.032). *p16 *gene hypermethylation only existed in 15 (32.6%) of 46 stage I carcinomas and none of the 14 stages II-IV carcinomas was positive (*P *= 0.014). But, the association between *p16 *gene hypermethylation and early-stage tumor was not confirmed by multivariate analysis (*P *= 0.748).

In ovarian cancer (Table 4), hypermethylation of *BRCA1 *was detected at a significantly higher frequency in serous carcinomas than in tumors of the other histological types (35.3% versus 6.2%, *P *= 0.015], whereas, *PTEN *were more frequently detected in mucinous carcinoma than in tumors of the other histological types (30% versus 2.6%, *P *= 0.023). Hypermethylation of *p16 *gene was detected in 33.3% (9/27) of serous or mucinous carcinomas, but not in any of the tumors of other histological types (*P *< 0.001). Additionally, *p14 *hypermethylation was associated with low-grade tumor (40.9% in G1 + G2 versus 12.0% in G3, *P *= 0.023) and early-stage cancer (37.5% in stage I+II versus 12.0% in stage III+IV, *P *= 0.038). Hypermethylation of *PTEN *was also associated with low-grade tumor, whereas, *BRCA1 *was associated with high-grade tumor (*P *= 0.026, 0.033 respectively). However, only the association between *BRCA1 *gene hypermethylation and serous carcinoma was confirmed by multivariate analysis (*P *= 0.045). More cases are needed for the confirmation of the other findings.

### Methylation-based prediction for therapeutic outcome and prognosis

Among 127 cervical cancer patients, there were a total of 31 deaths and 8 patients defaulted follow-up at 3 to 22 months with a median of 11.6 months. The remaining 88 patients had no evidence of disease on last follow-up at 3 to 153 months with a median of 83.7 months.

In survival analysis, the mean of overall survival (OS) was 114.3 months (95% confidence interval: 102.8–125.8 months). Using univariate survival analysis, FIGO stage was significantly associated with OS with estimated 5 year survival of 89% for stage I, 72% for stage II and 40% for stage III and IV (log rank test, *P *< 0.001). Modalities of treatment also was significantly associated with OS with estimated 5 year survival of 93% for patients treated by radical surgery, 86% for patients treated by radical surgery and radiotherapy and 66% for those treated by radiotherapy with or without chemotherapy (log-rank test, *P *= 0.022). Women younger than 51 had better survival than older women (5 year survival rate 83 vs 67%, log-rank test *P *= 0.015).

No significant association of survival was found in relation to grade 1 vs grades 2 & 3 (*P *= 0.066), histology types, methylation status of *DAPK *(*P *= 0.16), *MGMT *(*P *= 0.504), *p16 *(*P *= 0.258) and *PTEN *(*P *= 0.541). On multivariate analysis using Cox forward logistic regression with age, grade, stage, histology types, methylation status of *DAPK*, *MGMT*, *p16 *and *PTEN*, treatment modalities, only stage (*P *= 0.003, odds ratio = 1.9) remained as independent significant factor.

To evaluate the role of CpG island hypermethylation in the prediction of response to radiotherapy, 63 cervical cancer patients underwent primary radiotherapy were analyzed. Tumors were divided into radiosensitive and radioresistant groups based on the histological findings of residual tumor cells in the cervical biopsy specimens taken after the completion of external radiotherapy and brachytherapy. If there are no residual viable tumor cells in cervical biopsies, the tumor is defined as radiosensitive. Forty-four tumors were radiosensitive and 19 tumors were radioresistant. Tumors with methylation for any of the four genes were more sensitive to radiotherapy than tumors without (76.6% vs 50.0%, *P *= 0.045) (Table 2).

In endometrial cancer and ovarian cancer, no significant association was found between CpG island hypermethylation and overall survival. Using univariate survival analysis, none of the methylation status reached *P *< 0.05 (log-rank test). Larger sample size is needed for further confirmation.

## Discussion

### Rapid DNA methylation analysis by pooled DNA approach and MSP

Previous methods for DNA methylation analysis, such as Southern-blot analysis, bisulfite genomic DNA sequencing, and restriction enzyme digestion, require large amounts of DNA. The number of CpG islands investigated and sample sizes are also limited. Recently, some high-throughput approaches are developed for the large-scale analysis of multiple CpG islands including restriction landmark genomic scanning, differential methylation hybridization [17], and methylation specific oligonucleotide microarray [18]. However, there are some practical problems with these methods in dealing with clinical specimens. The discriminative ability of each probe is hampered by its different hybridization efficiency between methylated and unmethylated alleles. Moreover, these techniques need the most advanced chip technology and informatics approaches, which might not be widely available.

Compared to bisulfite sequencing, MSP is much simpler, requires less time and avoids the use of expensive sequencing reagents. Furthermore, simultaneous detection of unmethylated and methylated products in a single sample by MSP confirms the integrity of DNA as a template for PCR and allows a semi-quantitative assessment of allele types. Thus, in the present study, the pooled DNA approach and MSP were employed to screen those loci with aberrant methylation in tumors by comparing the methylation status in pooled tumor DNA and normal DNA. Only those loci with aberrant methylation in tumor were recognized as the potential epigenetic markers. Those loci with the same methylation status (presence or absence of methylated allele) in tumor and normal DNA pools were excluded for further study. In the present study, DNA methylation profiles in the three gynecologic cancers were compared easily and rapidly using the method introduced here.

### Differential DNA methylation profiles exist in gynecologic cancers

Specific pattern of CpG island hypermethylation existing in each human cancer was first reported by Costello et al. [5], and confirmed by Esteller et al. [6]. In the present study, 34 loci were randomly chosen for investigation. Those genes involve in many cellular pathways and have critical biological functions. Multiple genes were aberrantly methylated in the three cancers; in which some genes have been confirmed in other laboratories studying a single tumor type, whereas, others have not been reported before [15,16] and [19-25]. For each cancer type, aberrant methylation for several pathways happened simultaneously. For example, genes involved in cell cycle (*p16*), apoptosis (*DAPK*), cell adherence (*CDH1*), DNA repair (*MGMT*), and APC/β-cateninroute (*APC*) were found to be aberrantly methylated in cervical cancer. However, the profile of CpG island hypermethylation for the 34 loci differed among the three types of gynecologic cancers (Figure 2).

Hypermethylation of the *p16 *gene has been suggested to be a shared epigenetic alteration in multiple human cancers [6]. It was also observed across the three gynecologic cancers in the present study. The association between *DAPK *hypermethylation and SCC has been reported in our previous study [16] other studies [20,24]. In this study, aberrant methylation for the *DAPK *gene was only observed in cervical cancer, of which 80% of the samples were SCC. It was not detected in endometrial and ovarian cancers, both of which are AC. Thus, *DAPK *gene is selectively methylated in the development of SCC. *hMLH1 *is one of the DNA mismatch repair genes. Inactivation of *hMLH1 *results in microsatellite instability (MSI) in tumors [25]. MSI is predominant in tumors associated with HNPCC including endometrial cancer. Our previous study has found that a higher frequency of MSI was detected in endometrial cancer than in ovarian and cervical cancers (unpublished data), which could explain the presence of aberrant methylation of *hMLH1 *gene in endometrial cancer, but absence in cervical cancer and ovarian cancer. Thus, *hMLH1 *is susceptible to methylation in the development of endometrial cancer. *BRCA1 *is critical in the development of breast and ovarian cancers. Germline mutations account for most of the cases with hereditary breast and/or ovarian cancers [26]. However, in sporadic cancer, promoter hypermethylation, not somatic mutation, is the cause for *BRCA1 *inactivation [27]. In the present study, *BRCA1 *was specifically methylated in ovarian cancer, but not in the other two cancers. These observations have been confirmed in other laboratories studying a single tumor type. Thus, our results consistently demonstrate that differential DNA methylation profiles exist in the three gynecologic cancers.

Finally, we would like to raise a query concerning the FHIT gene. Our own result indicates that methylated alleles were found in all tumor and normal DNA (Figure 1) i.e. FHIT methylation was not tumor-specific. In contrast, it has been demonstrated that aberrant methylation of FHIT occurred specifically in lung and breast cancers [28]. However, in a study of FHIT methylation in cervical cancer, the methylation pattern identified by bisulfite genomic sequencing did not correlate with the MSP [29]. The authors suggested that false-positive results might be resulted from mispriming in the MSP assay. In this study, we used the primers exactly the same as those published information. Thus, one should be cautious when using biomarker for cancer. The recent publications on frequencies of FHIT methylation in human cancer have been tabulated [see Additional file 2] as references for those readers particularly interested in studying FHIT.

### Clinical implications of CpG island hypermethylation in gynecologic cancers

Esteller et al. [6] analyzed a series of promoter CpG island hypermethylation changes for 12 genes in DNA from over 600 primary tumor samples representing 15 major tumor types and concluded that a panel of three to four markers could define an abnormality in 70–90% of each cancer type. Thus, in the present study, 4 markers were chosen from all genes with aberrant methylation in each of the three cancers to determine the incidence of methylation in that particular cancer type and to define their role in cancer diagnosis and prognosis. Hypermethylation in any of the four genes investigated in each cancer type was detected in 50–70% of the patients. In each cancer type, methylation frequency varied among the four genes investigated, which indicated that CpG islands might differ in their susceptibility to de novo methylation.

Furthermore, association analysis demonstrated that hypermethylation of particular CpG islands was correlated with clinico-pathological parameters (tumor histology, grade or stage) and treatment response, which produced distinct epigenetic signatures for particular tumor subtypes. Thus, this epigenetic event has the potential to be used as a molecular marker for cancer diagnosis and prognosis in gynecologic cancers. The most significant finding is the *DAPK *gene hypermethylation in cervical cancer. *DAPK*, a pro-apoptotic serine/threonine kinase, involves in apoptosis and plays a role in tumor pathogenesis and metastasis when inactivated [30,31]. Inactivation of *DAPK*, mainly by promoter hypermethylation, has been demonstrated in some tumor types and found to be associated with aggressive and metastatic phenotype [32-35]. In cervical cancer, the correlation between *DAPK *gene hypermethylation and SCC demonstrated by other studies was further confirmed in this study. Our in-vitro study has shown that methylated promoter of *DAPK *was present in the radio-resistant cervical cancer cell line, SiHa; while absent in the radio-sensitive cell line, C4–1 (unpublished data). Moreover, the expression of *DAPK *was down regulated in SiHa cell line, but reactivated by exposure to the demethylation reagent (5-Aza-2' deoxycytidine), which was consistent to the findings by Narayan et al. [24]. Furthermore, our recent study has found that *DAPK *gene hypermethylation was detected in 50% of plasma samples of cervical cancer patients [16]. Thus, *DAPK *gene hypermethylation might be a valuable marker for tumor diagnosis, and evaluation of treatment outcome in cervical cancer. Because of its high frequency of methylation in cervical cancer, it might also be potentially used as a therapeutic target for cervical cancer treatment.

## Conclusion

Pooled DNA combined with MSP provides a useful approach for rapid methylation analysis of a large number of genes in multiple cancer types. CpG island hypermethylation is a frequent event in the development of the three gynecologic cancers. Differential methylation profiles exist in the three cancers and their subtypes. More studies using a much larger sample size are needed to further define the potential role of methylated DNA marker in cancer management.

## Competing interests

The author(s) declare that they have no competing interests.

## Authors' contributions

HJY performed most of the MSP analysis and prepared the draft of the manuscript. YW and PCKT did the DNA extraction and the initial MSP conditions testing. VWSL performed some of the MSP analysis, literature search and the preparing of the final manuscript. HYSN involved in the collection of samples and the analysis of the correlation between experimental and clinical data.

## Pre-publication history

The pre-publication history for this paper can be accessed here:



## Supplementary Material

Additional File 1 Primer sequence, genomic position, MSP condition and product size. The file contains the information on the primer sequences, genomic positions, MSP conditions and product sizes of the 34 loci analyzed in the present study. Click here for file

Additional File 2 Frequencies of FHIT gene methylation in various normal and tumor tissues of human cancers. The file contains the information on the frequencies of the FHIT gene methylation in various normal and tumor tissues of human cancers published in the last few years. Click here for file

## Figures and Tables

**Table 1:** Clinical characteristics of all patients with gynecologic cancers.

	Cervical cancer	Endometrial cancer	Ovarian cancer
Cases	127	60	50
Mean age (years)	51.9 (22–101)	58.2 (23–81)	48.8 (26–79)
FIGO stage			
I	61	46	15
II	46	6	9
III	17	6	20
IV	3	2	6
Grade			
G1	40	35	6
G2	53	17	16
G3	31	8	26
Unclear	3		2
Histological type			
Squamous	98		
Adenocarcinoma	29		
Serous			17
Mucinous			10
Endometrioid		60	11
Clear cell			6
Others			6
Treatment			
ST	32	38	6
RT	44		
CT			
ST+RT	19	21	
ST+CT			41
RT+CT+/- ST	30	1	3^a^
No treatment	2		

ST: surgery; RT: radiotherapy; CT: chemotherapy

^a^Those three patients have double primary cancers.

**Table 2:** Association of CpG island hypermethylation and clinico-pathological parameters in cervical cancer.

	All subjects	DAPK		p16		MGMT		PTEN		Any loci	
						
		+	-	+	-	+	-	+	-	+	-
Cases	127	72	55	32	95	21	106	20	107	92	35
Mean age (yrs)	51.9	52.8	50.7	53.5	51.4	49.8	52.3	52.9	51.7	53.0	48.9
P value(t-test)			0.422		0.484		0.463		0.734		0.152
Stage											
I	61	31	30	19	42	14	47	11	50	41	20
II	46	27	19	11	35	7	39	7	39	36	10
III-IV	20	14	6	2	18	0	20	2	18	15	5
P value(χ^2 ^test)			0.305		0.162		0.030^c^		0.688		0.431
Grade^a^											
G1	40	25	15	13	27	7	33	8	32	31	9
G2–3	84	46	38	18	66	13	71	12	72	60	24
P value(χ^2 ^test)			0.416		0.183		0.775		0.419		0.475
Histological type											
SCC	98	62	36	24	74	16	82	10	88	74	24
AC	29	10	19	8	21	5	24	10	19	18	11
P value(χ^2 ^test)			0.006		0.736		0.907		0.002		0.155
Response to radiotherapy^b^											
Radiosensitive	44	30	14	12	32	8	36	5	39	36	8
Radioresistant	19	9	10	3	16	2	17	3	16	11	8
P value(χ^2 ^test)			0.118		0.326		0.445		0.628		0.045

^a^WHO grading is not available for three cases.

^b^A total of 63 cases received primary radiotherapy and had the data of response to radiotherapy. ^c^Comparison was made between stages I+II and stages III+IV.

**Table 3:** Association of CpG island hypermethylation and clinico-pathological parameters in endometrial cancer.

	All subjects	CDH13		APC		p16		hMLH1		Any loci	
						
		+	-	+	-	+	-	+	-	+	-
Case	60	25	35	21	39	15	45	8	52	40	20
Mean age (yrs)	58.2	59.2	57.5	58.4	58.1	59.3	57.9	62.2	57.6	59.3	56.0
P value (T-test)			0.619		0.944		0.724		0.377		0.38
Stage											
I	46	20	26	17	29	15	31	6	40	35	11
II-IV	14	5	9	4	10	0	14	2	12	5	9
P value(χ^2 ^test)			0.606		0.565		0.014		0.905		0.005
Grade											
G1	35	12	23	10	25	11	24	1	34	22	13
G2–3	25	13	12	11	14	4	21	7	18	18	7
P value(χ^2 ^test)			0.17		0.217		0.174		0.007		0.459

**Table 4:** Association of CpG island hypermethylation and clinico-pathological parameters in ovarian cancer.

	All Subjects	p14		BRCA1		PTEN		p16		Any loci	
					
		+	-	+	-	+	-	+	-	+	-
Cases	49	12	37	8	41	4	45	9	40	25	24
Mean age (yrs)	48.8	47.6	49.2	44.0	49.8	65.1	47.4	52.6	48.0	47.8	49.9
P value (T-test)			0.7		0.239		0.006		0.321		0.559
Stage											
I-II	24	9	15	2	22	2	22	4	20	12	12
III-IV	25	3	22	6	19	2	23	5	20	13	12
P value (χ^2 ^test)			0.038		0.138		0.966		0.763		0.889
Grade^a^											
G1–2	22	9	13	1	21	4	18	6	16	13	9
G3	25	3	22	7	18	0	25	3	22	12	13
P value (χ^2 ^test)			0.023		0.033		0.026		0.184		0.447
Histological type	49										
Serous	17	2	15	6	11	1	16	3	14	11	6
Mucinous	10	3	7	1	9	3	7	6	4	7	3
Endometrioid	10	3	7	1	9	0	10	0	10	3	7
Clear-cell	6	3	3	0	6	0	6	0	6	3	3
Others	6	1	5	0	6	0	6	0	6	1	5
P value (χ^2 ^test]			0.08^b^		0.015^c^		0.023^d^		<0.001^e^		0.015^e^

^a^Who grading was not available for 2 cases.

^b^Comparison was made between mucinous/endometrioid/clear-cell and the other types.

^c^Comparison was made between serous and the other types.

^d^Comparison was made between mucinous and the other types.

^e^Comparison was made between serous/mucinous and the other types.
