# Supplementary material for: Differential DNA methylation profiles in gynecological cancers and correlation with clinico-pathological data
Source: BMC Cancer. 2006 Aug 23;6:212. doi: 10.1186/1471-2407-6-212 (PMC1560388; doi:10.1186/1471-2407-6-212)
Supplement: Additional File 1 — Primer sequence, genomic position, MSP condition and product size. The file contains the information on the primer sequences, genomic positions, MSP conditions and product sizes of the 34 loci analyzed in the present study. [file 1471-2407-6-212-S1.doc]

**Additional file 1:**

**Primer sequence, genomic position, MSP condition and product size**

| Gene name | Primer sequence | Genomic position**^*^** | Annealing temp. (°C) | Product size (bp) | Reference |
| --- | --- | --- | --- | --- | --- |
|  |  |  |  |  |  |
| ALX3-Uf | 5’-GGTGTTTATAGGTGGTGTGGGTAGT | -67 | 60 | 121 | [1] |
| ALX3-Ur | 5’-ATAAAAACCAAATACAAACCCCACACA |  |  |  |  |
| ALX3-Mf | 5’-GTTTATAGGCGGCGCGGGTAGC | -64 | 65 | 114 |  |
| ALX3-Mr | 5’-AAACCGAATACAAACCCCACGCG |  |  |  |  |
|  |  |  |  |  |  |
| APC-Uf | 5’-AATTTGTTGGATGTGGATTAGGGT | -118 | 60 | 89 | [2] |
| APC-Ur | 5’-AACCTCATATCAATCACATACA |  |  |  |  |
| APC-Mf | 5’-CGTTGGATGCGGATTAGGGC | -115 | 60 | 84 |  |
| APC-Mr | 5’-CCTCATATCGATCACGTACG |  |  |  |  |
|  |  |  |  |  |  |
| AR-Uf | 5’-TGTTTTTTTTGAGATTTTG | -9 | 43 | 213 | [3] |
| AR-Ur | 5’-CAAACAACAACTTCAAAACCA |  |  |  |  |
| AR-Mf | 5’-CGTTTTTTTCGAGATTTCG | -9 | 50 | 213 |  |
| AR-Mr | 5’-CGAACGACGACTTCGAAACCG |  |  |  |  |
|  |  |  |  |  |  |
| BRCA1-Uf | 5’’TTGGTTTTTGTGGTAATGGAAAAGTGT | +16 | 56 | 86 | [4] |
| BRCA1-Ur | 5’’CAAAAAATCTCAACAAACTCACACCA |  |  |  |  |
| BRCA1-Mf | 5’’TCGTGGTAACGGAAAAGCGC | +23 | 56 | 75 |  |
| BRCA1-Mr | 5’’AAATCTCAACGAACTCACGCCG |  |  |  |  |
|  |  |  |  |  |  |
| BRCA2-Uf | 5’’ATTAGGTGGTAGAGGTGGAGTT | +19 | 56 | 142 | [5] |
| BRCA2-Ur | 5’’CCAAAATAAACTAACAAAAACCA |  |  |  |  |
| BRCA2-Mf | 5’’GCGGTAGAGGCGGAGTC | +24 | 56 | 136 |  |
| BRCA2-Mr | 5’’CGAAATAAACTAACAAAAACCG |  |  |  |  |
|  |  |  |  |  |  |
| CACNA1G-Uf | 5’-GTTTTTTTTTGGATTTTTGTTTTTTG | -408 | 60 | 129 | [6] |
| CACNA1G-Ur | 5’-TTTATTCCAACTTCTTCACTTCA |  |  |  |  |
| CACNA1G-Mf | 5’-GTTTTTTCGGGGCGGTTTC | -361 | 62 | 78 |  |
| CACNA1G-Mr | 5’-TTCCGACTTCTTCGCTTCG |  |  |  |  |
|  |  |  |  |  |  |
| Casp8-Uf | 5’–TAGGGGATTTGGAGATTGTGA | -251 | 58 | 321 | [7] |
| Casp8-Ur | 5’–CCATATATCTACATTCAAAACAA |  |  |  |  |
| Casp8-Mf | 5’–TAGGGGATTCGGAGATTGCGA | -572 | 60 | 320 |  |
| Casp8-Mr | 5’–CGTATATCTACATTCAAAACGA |  |  |  |  |
|  |  |  |  |  |  |
| CDH1-Uf | 5’-TAATTTTAGGTTAGAGGGTTATTGT | -210 | 53 | 97 | [8-11] |
| CDH1-Ur | 5’-CACAACCAATCAACAACACA |  |  |  |  |
| CDH1-Mf | 5’-TTAGGTTAGAGGGTTATCGCGT | -182 | 57 | 116 |  |
| CDH1-Mr | 5’-TAACTAAAAATTCACCTACCGAC |  |  |  |  |
|  |  |  |  |  |  |
| CDH13-Uf | 5’-TTGTGGGGTTTGTTTTTTGT | -267 | 62 | 243 | [7] |
| CDH13-Ur | 5’-AACATTTTCATTCATACACACA |  |  |  |  |
| CDH13-Mf | 5’-TCGCGGGGTTCGTTTTTCGC | -267 | 62 | 243 |  |
| CDH13-Mr | 5’-GACGTTTTCATTCATACACGCG |  |  |  |  |
|  |  |  |  |  |  |
| DAPK1-Uf | 5’-GGAGGATAGTTGGATTGAGTTAATGTT | -332 | 60 | 106 | [9-12] |
| DAPK1-Ur | 5’-CAAATCCCTCCCAAACACCAA |  |  |  |  |
| DAPK1-Mf | 5’-GGATAGTCGGATCGAGTTAACGTC | -332 | 60 | 98 |  |
| DAPK1-Mr | 5’-CCCTCCCAAACGCCG |  |  |  |  |
|  |  |  |  |  |  |
| FHIT-Uf | 5’-TTGGGGTGTGGGTTTGGGTTTTTATG |  | 64 | 74 | [13] |
| FHIT-Ur | 5’-CATAAACAACACCAACCCCACTA |  |  |  |  |
| FHIT-Mf | 5’-TTGGGGCGCGGGTTTGGGTTTTTACGC |  | 64 | 74 |  |
| FHIT-Mr | 5’-CGTAAACGACGCCGACCCCACTA |  |  |  |  |
|  |  |  |  |  |  |
| HIC-1-Uf | 5’-TTGGGTTTGGTTTTTGTGTTTTG | -617 | 60 | 118 | [2] |
| HIC-1-Ur | 5’-CACCCTAACACCACCCTAAC |  |  |  |  |
| HIC-1-Mf | 5’-TCGGTTTTCGCGTTTTGTTCGT | -611 | 60 | 95 |  |
| HIC-1-Mr | 5’-AACCGAAAACTATCAACCCTCG |  |  |  |  |
|  |  |  |  |  |  |
| hMLH1-Uf | 5’’TTTTGATGTAGATGTTTTATTAGGGTTGT | -654 | 60 | 124 | [8,14] |
| hMLH1-Ur | 5’’ACCACCTCATCATAACTACCCACA |  |  |  |  |
| hMLH1-Mf | 5’’ACGTAGACGTTTTATTAGGGTCGC | -655 | 60 | 112 |  |
| hMLH1-Mr | 5’’CCTCATCGTAACTACCCGCG |  |  |  |  |
|  |  |  |  |  |  |
| hMSH2-Uf | 5’’GGTTGTTGTGGTTGGATGTTGTTT | -42 | 60 | 137 | [14] |
| hMSH2-Ur | 5’’CAACTACAACATCTCCTTCAACTACACCA |  |  |  |  |
| hMSH2-Mf | 5’’TCGTGGTCGGACGTCGTTC | -37 | 62 | 132 |  |
| hMSH2-Mr | 5’’CAACGTCTCCTTCGACTACACCG |  |  |  |  |
|  |  |  |  |  |  |
| hMSH3-Uf | 5’’GGTTTGTGTTTTTTGTTAGGTTTTGTT | +202 | 58 | 96 | [15] |
| hMSH3-Ur | 5’’CTAAAAACAACAAAACCACCCAACA |  |  |  |  |
| hMSH3-Mf | 5’’CGTTTTTCGTTAGGTTTTGTCGTC | +208 | 58 | 82 |  |
| hMSH3-Mr | 5’’AACGAAACCGCCCGACG |  |  |  |  |
|  |  |  |  |  |  |
| MGMT-Uf | 5’-TTTGTGTTTTGATGTTTGTAGGTTTTTGT | -46 | 59 | 93 | [8-11] |
| MGMT-Ur | 5’-AACTCCACACTCTTCCAAAAACAAAACA |  |  |  |  |
| MGMT-Mf | 5’-TTTCGACGTTCGTAGGTTTTCGC | +26 | 59 | 81 |  |
| MGMT-Mr | 5’-GCACTCTTCCGAAAACGAAACG |  |  |  |  |
|  |  |  |  |  |  |
| MINT1-Uf | 5’-GGGGTTGAGGTTTTTTGTTAGT |  | 64 | 117 | [6,10,11] |
| MINT1-Ur | 5’-TTCACAACCTCAAATCTACTTCA |  |  |  |  |
| MINT1-Mf | 5’-GGGTTGAGGTTTTTTGTTAGC |  | 64 | 102 |  |
| MINT1-Mr | 5’-CTACTTCGCCTAACCTAACG |  |  |  |  |
|  |  |  |  |  |  |
| MINT2-Uf | 5’-GGTGTTGTTAAATGTAAATAATTTG |  | 58 | 88 | [6,10,11] |
| MINT2-Ur | 5’-AAAAAAAAACACCTAAAACTCA |  |  |  |  |
| MINT2-Mf | 5’-AATCGAATTTGTCGTCGTTTC |  | 60 | 88 |  |
| MINT2-Mr | 5’-AAATAAATAAATAAAAAAAAACGCG |  |  |  |  |
|  |  |  |  |  |  |
| MINT31-Uf | 5’-GAATTGAGATGATTTTAATTTTTTGT |  | 64 | 105 | [6,10,11] |
| MINT31-Ur | 5’-CTAAAACCATCACCCCTAAACA |  |  |  |  |
| MINT31-Mf | 5’-TTGAGACGATTTTAATTTTTTGC |  | 62 | 100 |  |
| MINT31-Mr | 5’-AAAACCATCACCCCTAAACG |  |  |  |  |
|  |  |  |  |  |  |
| MINT32-Uf | 5’-GAGTGGTTAGAGGAATTTAGGT |  | 62 | 133 | [6,11] |
| MINT32-Ur | 5’-CTAAAAAAACAAACAAAACATCCA |  |  |  |  |
| MINT32-Mf | 5’-GTGGTTAGAGGAATTTAGGC |  | 64 | 126 |  |
| MINT32-Mr | 5’-AAAACGAACGAAACGTCCG |  |  |  |  |
|  |  |  |  |  |  |
| P14ARF-Uf | 5’-TTTTTGGTGTTAAAGGGTGGTGTAGT | +101 | 60 | 132 | [10,11] |
| P14ARF-Ur | 5’-CACAAAAACCCTCACTCACAACAA |  |  |  |  |
| P14ARF-Mf | 5’-GTGTTAAAGGGCGGCGTAGC | +107 | 60 | 122 |  |
| P14ARF-Mr | 5’-AAAACCCTCACTCGCGACGA |  |  |  |  |
|  |  |  |  |  |  |
| P15-Uf | 5’-TGTGATGTGTTTGTATTTTGTGGTT | -318 | 60 | 154 | [9,16] |
| P15-Ur | 5’-CCATACAATAACCAAACAACCAA |  |  |  |  |
| P15-Mf | 5’-GCGTTCGTATTTTGCGGTT | -312 | 60 | 148 |  |
| P15-Mr | 5’-CGTACAATAACCGAACGACCGA |  |  |  |  |
|  |  |  |  |  |  |
| P16ink4a-Uf | 5’’TTATTAGAGGGTGGGGTGGATTGT | -80 | 60 | 151 | [9,10,12,16] |
| P16ink4a-Ur | 5’’CAACCCCAAACCACAACCATAA |  |  |  |  |
| P16ink4a-Mf | 5’’TTATTAGAGGGTGGGGCGGATCGC | -80 | 60 | 150 |  |
| P16ink4a-Mr | 5’’GACCCCGAACCGCGACCGTAA |  |  |  |  |
|  |  |  |  |  |  |
| P73-Uf | 5’’AGGGGATGTAGTGAAATTGGGGTTT | +1 | 59 | 71 | [16,17] |
| P73-Ur | 5’’CCATCACAACCCCAAACATCA |  |  |  |  |
| P73-Mf | 5’’GGACGTAGCGAAATCGGGGTTC | +4 | 68 | 67 |  |
| P73-Mr | 5’’CGTCGCAACCCCGAACATCG |  |  |  |  |
|  |  |  |  |  |  |
| PTEN-Uf | 5’’GTGTTGGTGGAGGTAGTTGTTT | -285 | 62 | 162 | [10,18] |
| PTEN-Ur | 5’’ACCACTTAACTCTAAACCACAACCA |  |  |  |  |
| PTEN-Mf | 5’’TTCGTTCGTCGTCGTCGTATTT | -329 | 62 | 206 |  |
| PTEN-Mr | 5’’GCCGCTTAACTCTAAACCGCAACCG |  |  |  |  |
|  |  |  |  |  |  |
| RAR-Beta2-Uf | 5’-TTAGTAGTTTGGGTAGGGTTTATT | -69 | 59 | 233 | [9] |
| RAR-Beta2-Ur | 5’-CCAAATCCTACCCCAACA |  |  |  |  |
| RAR-Beta2-Mf | 5’-GGTTAGTAGTTCGGGTAGGGTTTATC | -71 | 59 | 235 |  |
| RAR-Beta2-Mr | 5’-CCGAATCCTACCCCGACG |  |  |  |  |
|  |  |  |  |  |  |
| RASSF1A-Uf | 5’-GGTTTTGTGAGAGTGTGTTTAG | -73 | 59 | 169 | [19] |
| RASSF1A-Ur | 5’-CACTAACAAACACAAACCAAAC |  |  |  |  |
| RASSF1A-Mf | 5’-GGGTTTTGCGAGAGCGCG | -73 | 64 | 169 |  |
| RASSF1A-Mr | 5’-GCTAACAAACGCGAACCG |  |  |  |  |
|  |  |  |  |  |  |
| RB1-Uf | 5’-GGGAGTTTTGTGGATGTGAT | -286 | 55 | 173 | [7,20] |
| RB1-Ur | 5’-ACATCAAAACACACCCCA |  |  |  |  |
| RB1-Mf | 5’-GGGAGTTTCGCGGACGTGAC | -286 | 55 | 173 |  |
| RB1-Mr | 5’-ACGTCGAAACACGCCCCG |  |  |  |  |
|  |  |  |  |  |  |
| RIZ1-Uf | 5’-TGGTGGTTATTGGGTGATGGT | -165 | 60 | 175 | [21] |
| RIZ1-Ur | 5’-ACTATTTCACCAACCCCAACA |  |  |  |  |
| RIZ1-Mf | 5’-GTGGTGGTTATTGGGCGACGGC | -166 | 68 | 176 |  |
| RIZ1-Mr | 5’-GCTATTTCGCCGACCCCGACG |  |  |  |  |
|  |  |  |  |  |  |
| STK11-Uf | 5’-GGATGAAGTTGATTTTGATTGGGTT | +792 | 55 | 122 | [22] |
| STK11-Ur | 5’-ACCCAATACAAAATCTACAAACCAACA |  |  |  |  |
| STK11-Mf | 5’-ACGAAGTTGATTTTGATCGGGTC | +794 | 58 | 117 |  |
| STK11-Mr | 5’-CGATACAAAATCTACGAACCGACG |  |  |  |  |
|  |  |  |  |  |  |
| THBS1-Uf | 5’-GTTTGGTTGTTGTTTATTGGTTG | -81 | 62 | 115 | [10-12] |
| THBS1-Ur | 5’-CCTAAACTCACAAACCAACTCA |  |  |  |  |
| THBS1-Mf | 5’-TGCGAGCGTTTTTTTAAAAGC | -42 | 62 | 74 |  |
| THBS1-Mr | 5’-TAAACTCGCAAACCAACTCG |  |  |  |  |
|  |  |  |  |  |  |
| TIMP-3-Uf | 5’-TTTTGTTTTGTTATTTTTTGTTTTTGGTTTT | +730 | 59 | 122 | [10-12] |
| TIMP-3-Ur | 5’-CCCCCCAAAAACCCCACCTCA |  |  |  |  |
| TIMP-3-Mf | 5’-CGTTTCGTTATTTTTTGTTTTCGGTTTTC | +733 | 59 | 116 |  |
| TIMP-3-Mr | 5’-CCGAAAACCCCGCCTCG |  |  |  |  |
|  |  |  |  |  |  |
| TMS1-Uf | 5’-GGTTGTAGTGGGGTGAGTGGT | -46 | 58 | 191 | [23-25] |
| TMS1-Ur | 5’-CAAAACATCCATAAACAACAACACA |  |  |  |  |
| TMS1-Mf | 5’-TTGTAGCGGGGTGAGCGGC | -44 | 58 | 196 |  |
| TMS1-Mr | 5’-AACGTCCATAAACAACAACGCG |  |  |  |  |
|  |  |  |  |  |  |
| VHL-Uf | 5’-GTTGGAGGATTTTTTTGTGTATGT | -185 | 60 | 165 | [7,8] |
| VHL-Ur | 5’-CCCAAACCAAACACCACAAA |  |  |  |  |
| VHL-Mf | 5’-TGGAGGATTTTTTTGCGTACGC | -183 | 60 | 158 |  |
| VHL-Mr | 5’-GAACCGAACGCCGCGAA |  |  |  |  |
|  |  |  |  |  |  |

Uf = unmethylated forward primer; Ur = unmethylated reverse primer

Mf = methylated forward primer; Mr = unmethylated reverse primer

**^*^**The 5’ positions of the sense unmethylated and methylated are numbered relative to the transcription start site of the gene concern.

**References**

1. [Wimmer K](http://www.ncbi.nlm.nih.gov/entrez/query.fcgi?db=pubmed&cmd=Search&itool=pubmed_Abstract&term="Wimmer+K"%5BAuthor%5D), [Zhu XX](http://www.ncbi.nlm.nih.gov/entrez/query.fcgi?db=pubmed&cmd=Search&itool=pubmed_Abstract&term="Zhu+Xx+XX"%5BAuthor%5D), [Rouillard JM](http://www.ncbi.nlm.nih.gov/entrez/query.fcgi?db=pubmed&cmd=Search&itool=pubmed_Abstract&term="Rouillard+JM"%5BAuthor%5D), [Ambros PF](http://www.ncbi.nlm.nih.gov/entrez/query.fcgi?db=pubmed&cmd=Search&itool=pubmed_Abstract&term="Ambros+PF"%5BAuthor%5D), [Lamb BJ](http://www.ncbi.nlm.nih.gov/entrez/query.fcgi?db=pubmed&cmd=Search&itool=pubmed_Abstract&term="Lamb+BJ"%5BAuthor%5D), [Kuick R](http://www.ncbi.nlm.nih.gov/entrez/query.fcgi?db=pubmed&cmd=Search&itool=pubmed_Abstract&term="Kuick+R"%5BAuthor%5D), [Eckart M](http://www.ncbi.nlm.nih.gov/entrez/query.fcgi?db=pubmed&cmd=Search&itool=pubmed_Abstract&term="Eckart+M"%5BAuthor%5D), [Weinhausl A](http://www.ncbi.nlm.nih.gov/entrez/query.fcgi?db=pubmed&cmd=Search&itool=pubmed_Abstract&term="Weinhausl+A"%5BAuthor%5D), [Fonatsch C](http://www.ncbi.nlm.nih.gov/entrez/query.fcgi?db=pubmed&cmd=Search&itool=pubmed_Abstract&term="Fonatsch+C"%5BAuthor%5D), [Hanash SM](http://www.ncbi.nlm.nih.gov/entrez/query.fcgi?db=pubmed&cmd=Search&itool=pubmed_Abstract&term="Hanash+SM"%5BAuthor%5D): **Combined restriction landmark genomic scanning and virtual genome scans identify a novel human homeobox gene, ALX3, that is hypermethylated in neuroblastoma.** [*Genes Chromosomes Cancer*](javascript:AL_get(this, 'jour', 'Genes Chromosomes Cancer.');) 2002, **33**:285-294.
2. Dong SM, Kim HS, Rha SH, Sidransky D: **Promoter Hypermethylation of Multiple Genes in Carcinoma of the Uterine Cervix.** *Clin Cancer Res* 2001, **7**:1982-1986.
3. Sasaki M, Tanaka Y, Perinchery G, Dharia A, Kotcherguina I, Fujimoto S, Dahiya R: **Methylation and Inactivation of Estrogen, Progesterone, and Androgen Receptors in Prostate Cancer. *J*** *Natl Cancer Inst* 2002, **94**:384-390.
4. Esteller M, Silva JM, Dominguez G, Bonilla F, Matias-Guiu X, Lerma E, Bussaglia E, Prat J, Harkes I.C, Repasky EA, Gabrielson E, Schutte M, Baylin SB, Herman JG: **Promoter Hypermethylation and BRCA1 Inactivation in Sporadic Breast and Ovarian Tumors.** *J Natl Cancer Inst* 2000, **92**:564-569.
5. [Gras E](http://www.ncbi.nlm.nih.gov/entrez/query.fcgi?db=pubmed&cmd=Search&itool=pubmed_Abstract&term="Gras+E"%5BAuthor%5D), [Cortes J](http://www.ncbi.nlm.nih.gov/entrez/query.fcgi?db=pubmed&cmd=Search&itool=pubmed_Abstract&term="Cortes+J"%5BAuthor%5D), [Diez O](http://www.ncbi.nlm.nih.gov/entrez/query.fcgi?db=pubmed&cmd=Search&itool=pubmed_Abstract&term="Diez+O"%5BAuthor%5D), [Alonso C](http://www.ncbi.nlm.nih.gov/entrez/query.fcgi?db=pubmed&cmd=Search&itool=pubmed_Abstract&term="Alonso+C"%5BAuthor%5D), [Matias-Guiu X](http://www.ncbi.nlm.nih.gov/entrez/query.fcgi?db=pubmed&cmd=Search&itool=pubmed_Abstract&term="Matias-Guiu+X"%5BAuthor%5D), [Baiget M](http://www.ncbi.nlm.nih.gov/entrez/query.fcgi?db=pubmed&cmd=Search&itool=pubmed_Abstract&term="Baiget+M"%5BAuthor%5D), [Prat J](http://www.ncbi.nlm.nih.gov/entrez/query.fcgi?db=pubmed&cmd=Search&itool=pubmed_Abstract&term="Prat+J"%5BAuthor%5D): **Loss of heterozygosity on chromosome 13q12-q14, BRCA-2 mutations and lack of BRCA-2 promoter hypermethylation in sporadic epithelial ovarian tumors.** [*Cance*](javascript:AL_get(this, 'jour', 'Cancer.');)*r* 2001, **92**:787-795.
6. [Ueki T, Toyota M, Sohn T, Yeo CJ, Issa JP, Hruban RH, Goggins M:](http://www.ncbi.nlm.nih.gov/entrez/query.fcgi?cmd=Retrieve&db=pubmed&dopt=Abstract&list_uids=10766168&query_hl=14&itool=pubmed_docsum) **Hypermethylation of multiple genes in pancreatic adenocarcinoma.** *Cancer Res* 2000, **60**:1835-1839.
7. [Yu J, Ni M, Xu J, Zhang H, Gao B, Gu J, Chen J, Zhang L, Wu M, Zhen S, Zhu J:](http://www.ncbi.nlm.nih.gov/entrez/query.fcgi?cmd=Retrieve&db=pubmed&dopt=Abstract&list_uids=12433278&query_hl=39&itool=pubmed_docsum) **Methylation profiling of twenty promoter-CpG islands of genes which may contribute to hepatocellular carcinogenesis.** *BMC Cancer* 2002, **2**:29.
8. [Chung WB](http://www.ncbi.nlm.nih.gov/entrez/query.fcgi?db=pubmed&cmd=Search&itool=pubmed_Abstract&term="Chung+WB"%5BAuthor%5D), [Hong SH](http://www.ncbi.nlm.nih.gov/entrez/query.fcgi?db=pubmed&cmd=Search&itool=pubmed_Abstract&term="Hong+SH"%5BAuthor%5D), [Kim JA](http://www.ncbi.nlm.nih.gov/entrez/query.fcgi?db=pubmed&cmd=Search&itool=pubmed_Abstract&term="Kim+JA"%5BAuthor%5D), [Sohn YK](http://www.ncbi.nlm.nih.gov/entrez/query.fcgi?db=pubmed&cmd=Search&itool=pubmed_Abstract&term="Sohn+YK"%5BAuthor%5D), [Kim BW](http://www.ncbi.nlm.nih.gov/entrez/query.fcgi?db=pubmed&cmd=Search&itool=pubmed_Abstract&term="Kim+BW"%5BAuthor%5D), [Kim JW](http://www.ncbi.nlm.nih.gov/entrez/query.fcgi?db=pubmed&cmd=Search&itool=pubmed_Abstract&term="Kim+JW"%5BAuthor%5D): **Hypermethylation of tumor-related genes in genitourinary cancer cell lines.** [*J Korean Med Sci*](javascript:AL_get(this, 'jour', 'J Korean Med Sci.');) 2001, **16**:756-761.
9. [Chan MW](http://www.ncbi.nlm.nih.gov/entrez/query.fcgi?db=pubmed&cmd=Search&itool=pubmed_Abstract&term="Chan+MW"%5BAuthor%5D), [Chan LW](http://www.ncbi.nlm.nih.gov/entrez/query.fcgi?db=pubmed&cmd=Search&itool=pubmed_Abstract&term="Chan+LW"%5BAuthor%5D), [Tang NL](http://www.ncbi.nlm.nih.gov/entrez/query.fcgi?db=pubmed&cmd=Search&itool=pubmed_Abstract&term="Tang+NL"%5BAuthor%5D), [Tong JH](http://www.ncbi.nlm.nih.gov/entrez/query.fcgi?db=pubmed&cmd=Search&itool=pubmed_Abstract&term="Tong+JH"%5BAuthor%5D), [Lo KW](http://www.ncbi.nlm.nih.gov/entrez/query.fcgi?db=pubmed&cmd=Search&itool=pubmed_Abstract&term="Lo+KW"%5BAuthor%5D), [Lee TL](http://www.ncbi.nlm.nih.gov/entrez/query.fcgi?db=pubmed&cmd=Search&itool=pubmed_Abstract&term="Lee+TL"%5BAuthor%5D), [Cheung HY](http://www.ncbi.nlm.nih.gov/entrez/query.fcgi?db=pubmed&cmd=Search&itool=pubmed_Abstract&term="Cheung+HY"%5BAuthor%5D), [Wong WS](http://www.ncbi.nlm.nih.gov/entrez/query.fcgi?db=pubmed&cmd=Search&itool=pubmed_Abstract&term="Wong+WS"%5BAuthor%5D), [Chan PS](http://www.ncbi.nlm.nih.gov/entrez/query.fcgi?db=pubmed&cmd=Search&itool=pubmed_Abstract&term="Chan+PS"%5BAuthor%5D), [Lai FM](http://www.ncbi.nlm.nih.gov/entrez/query.fcgi?db=pubmed&cmd=Search&itool=pubmed_Abstract&term="Lai+FM"%5BAuthor%5D), [To KF](http://www.ncbi.nlm.nih.gov/entrez/query.fcgi?db=pubmed&cmd=Search&itool=pubmed_Abstract&term="To+KF"%5BAuthor%5D): **Hypermethylation of multiple genes in tumor tissues and voided urine in urinary bladder cancer patients.** [*Clin Cancer Res*](javascript:AL_get(this, 'jour', 'Clin Cancer Res.');) 2002, **8**:464-470.
10. [Kang GH, Lee S, Kim WH, Lee HW, Kim JC, Rhyu MG, Ro JY:](http://www.ncbi.nlm.nih.gov/entrez/query.fcgi?cmd=Retrieve&db=pubmed&dopt=Abstract&list_uids=11891177&query_hl=22&itool=pubmed_DocSum) **Epstein-barr virus-positive gastric carcinoma demonstrates frequent aberrant methylation of multiple genes and constitutes CpG island methylator phenotype-positive gastric carcinoma.** Am J Pathol 2002, **160**:787-794.
11. [Lee S](http://www.ncbi.nlm.nih.gov/entrez/query.fcgi?db=pubmed&cmd=Search&itool=pubmed_Abstract&term="Lee+S"%5BAuthor%5D), [Kim WH](http://www.ncbi.nlm.nih.gov/entrez/query.fcgi?db=pubmed&cmd=Search&itool=pubmed_Abstract&term="Kim+WH"%5BAuthor%5D), [Jung HY](http://www.ncbi.nlm.nih.gov/entrez/query.fcgi?db=pubmed&cmd=Search&itool=pubmed_Abstract&term="Jung+HY"%5BAuthor%5D), [Yang MH](http://www.ncbi.nlm.nih.gov/entrez/query.fcgi?db=pubmed&cmd=Search&itool=pubmed_Abstract&term="Yang+MH"%5BAuthor%5D), [Kang GH](http://www.ncbi.nlm.nih.gov/entrez/query.fcgi?db=pubmed&cmd=Search&itool=pubmed_Abstract&term="Kang+GH"%5BAuthor%5D): **Aberrant CpG island methylation of multiple genes in intrahepatic cholangiocarcinoma.** [*Am J Pathol*](javascript:AL_get(this, 'jour', 'Am J Pathol.');) 2002, **161**:1015-1022.
12. [Kang GH, Shim YH, Jung HY, Kim WH, Ro JY, Rhyu MG:](http://www.ncbi.nlm.nih.gov/entrez/query.fcgi?cmd=Retrieve&db=pubmed&dopt=Abstract&list_uids=11306456&query_hl=9&itool=pubmed_DocSum) **CpG island methylation in premalignant stages of gastric carcinoma.** *Cancer Res* 2001, **61**:2847-2851.
13. Zöchbauer-Müller S, Fong KM, Maitra A, Lam S, Geradts J, Ashfaq R, Virmani AK, Milchgrub S, Gazdar AF, Minna JD: **5' CpG Island Methylation of the *FHIT* Gene Is Correlated with Loss of Gene Expression in Lung and Breast Cancer**. *Cancer Res* 2001, **61**:3581-3585.
14. [Herman JG](http://www.ncbi.nlm.nih.gov/entrez/query.fcgi?db=pubmed&cmd=Search&itool=pubmed_Abstract&term="Herman+JG"%5BAuthor%5D), [Umar A](http://www.ncbi.nlm.nih.gov/entrez/query.fcgi?db=pubmed&cmd=Search&itool=pubmed_Abstract&term="Umar+A"%5BAuthor%5D), [Polyak K](http://www.ncbi.nlm.nih.gov/entrez/query.fcgi?db=pubmed&cmd=Search&itool=pubmed_Abstract&term="Polyak+K"%5BAuthor%5D), [Graff JR](http://www.ncbi.nlm.nih.gov/entrez/query.fcgi?db=pubmed&cmd=Search&itool=pubmed_Abstract&term="Graff+JR"%5BAuthor%5D), [Ahuja N](http://www.ncbi.nlm.nih.gov/entrez/query.fcgi?db=pubmed&cmd=Search&itool=pubmed_Abstract&term="Ahuja+N"%5BAuthor%5D), [Issa JP](http://www.ncbi.nlm.nih.gov/entrez/query.fcgi?db=pubmed&cmd=Search&itool=pubmed_Abstract&term="Issa+JP"%5BAuthor%5D), [Markowitz S](http://www.ncbi.nlm.nih.gov/entrez/query.fcgi?db=pubmed&cmd=Search&itool=pubmed_Abstract&term="Markowitz+S"%5BAuthor%5D), [Willson JK](http://www.ncbi.nlm.nih.gov/entrez/query.fcgi?db=pubmed&cmd=Search&itool=pubmed_Abstract&term="Willson+JK"%5BAuthor%5D), [Hamilton SR](http://www.ncbi.nlm.nih.gov/entrez/query.fcgi?db=pubmed&cmd=Search&itool=pubmed_Abstract&term="Hamilton+SR"%5BAuthor%5D), [Kinzler KW](http://www.ncbi.nlm.nih.gov/entrez/query.fcgi?db=pubmed&cmd=Search&itool=pubmed_Abstract&term="Kinzler+KW"%5BAuthor%5D), [Kane MF](http://www.ncbi.nlm.nih.gov/entrez/query.fcgi?db=pubmed&cmd=Search&itool=pubmed_Abstract&term="Kane+MF"%5BAuthor%5D), [Kolodner RD](http://www.ncbi.nlm.nih.gov/entrez/query.fcgi?db=pubmed&cmd=Search&itool=pubmed_Abstract&term="Kolodner+RD"%5BAuthor%5D), [Vogelstein B](http://www.ncbi.nlm.nih.gov/entrez/query.fcgi?db=pubmed&cmd=Search&itool=pubmed_Abstract&term="Vogelstein+B"%5BAuthor%5D), [Kunkel TA](http://www.ncbi.nlm.nih.gov/entrez/query.fcgi?db=pubmed&cmd=Search&itool=pubmed_Abstract&term="Kunkel+TA"%5BAuthor%5D), [Baylin SB](http://www.ncbi.nlm.nih.gov/entrez/query.fcgi?db=pubmed&cmd=Search&itool=pubmed_Abstract&term="Baylin+SB"%5BAuthor%5D): **Incidence and functional consequences of hMLH1 promoter hypermethylation in colorectal carcinoma.** [*Proc Natl Acad Sci U S A*](javascript:AL_get(this, 'jour', 'Proc Natl Acad Sci U S A.');) 1998, **95**:6870-6875.
15. Esteller M, Catasus L, Matias-Guiu X, Mutter GL, Prat J, Baylin SB, Herman JG: ***hMLH1* Promoter Hypermethylation Is an Early Event in Human Endometrial Tumorigenesis.** *Am J Pathol* 1999, **155**:1767-1772.
16. Siu LLP, Chan JKC, Wong KF, Kwong YL: **Specific Patterns of Gene Methylation in Natural Killer Cell Lymphomas : p73 Is Consistently Involved.** *Am J Pathol* 2002, **160**:59-66.
17. [Liu M, Taketani T, Li R, Takita J, Taki T, Yang HW, Kawaguchi H, Ida K, Matsuo Y, Hayashi Y:](http://www.ncbi.nlm.nih.gov/entrez/query.fcgi?cmd=Retrieve&db=pubmed&dopt=Abstract&list_uids=11337015&query_hl=1&itool=pubmed_DocSum) **Loss of p73 gene expression in lymphoid leukemia cell lines is associated with hypermethylation.** *Leuk Res* 2001, **25**:441-447.
18. [Salvesen HB, MacDonald N, Ryan A, Jacobs IJ, Lynch ED, Akslen LA, Das S:](http://www.ncbi.nlm.nih.gov/entrez/query.fcgi?cmd=Retrieve&db=pubmed&dopt=Abstract&list_uids=11149415&query_hl=32&itool=pubmed_docsum) **PTEN methylation is associated with advanced stage and microsatellite instability in endometrial carcinoma.** *Int J Cancer* 2001, **91**:22-26.
19. Burbee DG, Forgacs E, Zöchbauer-Müller S, Shivakumar L, Fong K, Gao B, Randle D, Kondo M, Virmani A, Bader S, Sekido Y, Latif F, Milchgrub S, Toyooka S, Gazdar AF, Lerman MI, Zabarovsky E, White M, Minna JD: **Epigenetic Inactivation of RASSF1A in Lung and Breast Cancers and Malignant Phenotype Suppression**. *J Natl Cancer Inst* 2001, **93**:691-699.
20. [Simpson DJ, Hibberts NA, McNicol AM, Clayton RN, Farrell WE:](http://www.ncbi.nlm.nih.gov/entrez/query.fcgi?cmd=Retrieve&db=pubmed&dopt=Abstract&list_uids=10728677&query_hl=1&itool=pubmed_docsum) **Loss of pRb expression in pituitary adenomas is associated with methylation of the RB1 CpG island.** *Cancer Res* 2000, **60**:1211-1216.
21. [Du Y, Carling T, Fang W, Piao Z, Sheu JC, Huang S :](http://www.ncbi.nlm.nih.gov/entrez/query.fcgi?cmd=Retrieve&db=pubmed&dopt=Abstract&list_uids=11719434&query_hl=3&itool=pubmed_docsum) **Hypermethylation in human cancers of the RIZ1 tumor suppressor gene, a member of a histone/protein methyltransferase superfamily.** *Cancer Res* 2001, **61**:8094-8099.
22. [Esteller M, Avizienyte E, Corn PG, Lothe RA, Baylin SB, Aaltonen LA, Herman JG:](http://www.ncbi.nlm.nih.gov/entrez/query.fcgi?cmd=Retrieve&db=pubmed&dopt=Abstract&list_uids=10644993&query_hl=42&itool=pubmed_DocSum) **Epigenetic inactivation of LKB1 in primary tumors associated with the Peutz-Jeghers syndrome.** *Oncogene* 2000, **19**:164-168.
23. Guan X, Sagara J, Yokoyama T, Koganehira Y, Oguchi M, Saida T, Taniguchi S: **ASC/TMS1, a caspase-1 activating adaptor, is downregulated by aberrant methylation in human melanoma.** *Int J Cancer* 2003, **107**:202-208.
24. [Akahira J, Sugihashi Y, Ito K, Niikura H, Okamura K, Yaegashi N:](http://www.ncbi.nlm.nih.gov/entrez/query.fcgi?cmd=Retrieve&db=pubmed&dopt=Abstract&list_uids=14720325&query_hl=4&itool=pubmed_DocSum) **Promoter methylation status and expression of TMS1 gene in human epithelial ovarian cancer.**
    *Cancer Sci* 2004, **95**:40-43.
25. [Terasawa K, Sagae S, Toyota M, Tsukada K, Ogi K, Satoh A, Mita H, Imai K, Tokino T, Kudo R:](http://www.ncbi.nlm.nih.gov/entrez/query.fcgi?cmd=Retrieve&db=pubmed&dopt=Abstract&list_uids=15041718&query_hl=1&itool=pubmed_docsum) **Epigenetic inactivation of TMS1/ASC in ovarian cancer.** *Clin Cancer Res* 2004, **10**:2000-2006.
