# Supplementary material for: Differential DNA methylation profiles in gynecological cancers and correlation with clinico-pathological data
Source: BMC Cancer. 2006 Aug 23;6:212. doi: 10.1186/1471-2407-6-212 (PMC1560388; doi:10.1186/1471-2407-6-212)
Supplement: Additional File 2 — Frequencies of FHIT gene methylation in various normal and tumor tissues of human cancers. The file contains the information on the frequencies of the FHIT gene methylation in various normal and tumor tissues of human cancers published in the last few years. [file 1471-2407-6-212-S2.doc]

**Additional file 2:**

**Frequencies of the FHIT gene methylation in various normal and tumor tissues of human cancers**

| **Cancer type** | **Frequency in normal tissue and tissue type** | **Frequency in tumor tissue** | **Primer used** | **Reference** |
| --- | --- | --- | --- | --- |
| Acute myeloid leukemia | 0% (0/14)  Mononuclear blood cells | 14% (13/94) | Same as present study | [6] |
| Barrett’s oesophagus | 0% (0/32)  Normal squamous oesophageal epithelium and normal gastric epithelium | 3.7% (1/27) | MS-SSCA^a^ | [2] |
| Bladder cancer | 0% (0/7)  Non neoplastic adjacent tissues | 11% (3/27) | Same as present study | [5] |
| Breast cancer | 14% (4/28)  Non neoplastic adjacent tissues | 57% (16/28) | Same as present study | [5] |
| Cervical cancer | 0% (0/10) | 40% (16/40) | Not clear | [15] |
| Children intracranial ependymomas | 0% (0/3)  Adult corteses | 22% (6/27) | Same as present study | [10] |
| Esophageal cancer | NI^b^ | 33% (85/257) | Same as present study | [9] |
| Esophageal squamous cell carcinoma | 30% (14/47)  Corresponding noncancerous tissues from patients | 45% (21/47) | Same as present study | [8] |
| Granulosa cell tumors | 0% (0/3)  Normal tissue distant from lesion | 28% (7/25) | Same as present study | [3] |
| Intrahepatic cholangio-carcinomas | NI | 42% (8/19) | COBRA^c^ | [4] |
| Lung cancer | NI | 37% (11/30) | Same as present study | [14] |
| Lung cancer | 8.3% (2/24)  Non neoplastic adjacent tissues | 38% (9/24) | Same as present study | [5] |
| Multiple myeloma | NI | 44% (21/48) | Same as present study | [12] |
| Non small cell lung cancer | NI | 52.2% (117/224) | Same as present study | [11] |
| Non small cell Lung Cancer | NI | 21% (70/335) | Same as present study | [7] |
| Non small cell lung cancer | NI | 36% (43/120) | Same as present study | [13] |
| Primitive neuroectodermal tumor | 0% (0/7)  Cerebellar and Cerebral tissues | 22% (2/9) | Same as present study | [1] |

^a^MS-SSCA = methylation sensitive single-strand conformation analysis

^b^NI = Not Indicated in the report

^c^COBRA = combined bisulfite restriction analysis

**References**

1. [Chang Q, Pang JC, Li KK, Poon WS, Zhou L, Ng HK:](http://www.ncbi.nlm.nih.gov/entrez/query.fcgi?db=pubmed&cmd=Retrieve&dopt=AbstractPlus&list_uids=16311119&query_hl=9&itool=pubmed_DocSum) **Promoter hypermethylation profile of RASSF1A, FHIT, and sFRP1 in intracranial primitive neuroectodermal tumors.** *Hum Pathol* 2005, 36:1265-1272.
2. [Clement G, Braunschweig R, Pasquier N, Bosman FT, Benhattar J:](http://www.ncbi.nlm.nih.gov/entrez/query.fcgi?db=pubmed&cmd=Retrieve&dopt=AbstractPlus&list_uids=16278815&query_hl=9&itool=pubmed_DocSum) **Methylation of APC, TIMP3, and TERT: a new predictive marker to distinguish Barrett's oesophagus patients at risk for malignant transformation.** *J Pathol* 2006, **208**:100-107.
3. [Dhillon VS, Shahid M, Husain SA:](http://www.ncbi.nlm.nih.gov/entrez/query.fcgi?db=pubmed&cmd=Retrieve&dopt=AbstractPlus&list_uids=15574200&query_hl=9&itool=pubmed_DocSum) **CpG methylation of the FHIT, FANCF, cyclin-D2, BRCA2 and RUNX3 genes in Granulosa cell tumors (GCTs) of ovarian origin.** *Mol Cancer* 2004, **3**:33-40.
4. [Foja S, Goldberg M, Schagdarsurengin U, Dammann R, Tannapfel A, Ballhausen WG:](http://www.ncbi.nlm.nih.gov/entrez/query.fcgi?db=pubmed&cmd=Retrieve&dopt=AbstractPlus&list_uids=16343073&query_hl=9&itool=pubmed_DocSum) **Promoter methylation and loss of coding exons of the fragile histidine triad (FHIT) gene in intrahepatic cholangiocarcinomas.** *Liver Int* 2005, **25**:1202-1208.
5. [Iliopoulos D, Guler G, Han SY, Johnston D, Druck T, McCorkell KA, Palazzo J, McCue PA, Baffa R, Huebner K:](http://www.ncbi.nlm.nih.gov/entrez/query.fcgi?db=pubmed&cmd=Retrieve&dopt=AbstractPlus&list_uids=15674328&query_hl=9&itool=pubmed_DocSum) **Fragile genes as biomarkers: epigenetic control of WWOX and FHIT in lung, breast and bladder cancer.** *Oncogene* 2005, **24**:1625-1633.
6. [Iwai M, Kiyoi H, Ozeki K, Kinoshita T, Emi N, Ohno R, Naoe T:](http://www.ncbi.nlm.nih.gov/entrez/query.fcgi?db=pubmed&cmd=Retrieve&dopt=AbstractPlus&list_uids=15902282&query_hl=9&itool=pubmed_DocSum) **Expression and methylation status of the FHIT gene in acute myeloid leukemia and myelodysplastic syndrome.** *Leukemia* 2005, **19**:1367-1375.
7. [Kim JS, Kim JW, Han J, Shim YM, Park J, Kim DH:](http://www.ncbi.nlm.nih.gov/entrez/query.fcgi?db=pubmed&cmd=Retrieve&dopt=AbstractPlus&list_uids=16618724&query_hl=9&itool=pubmed_DocSum) **Cohypermethylation of p16 and FHIT promoters as a prognostic factor of recurrence in surgically resected stage I non-small cell lung cancer.** *Cancer Res* 2006, **66**:4049-4054.
8. [Kuroki T, Trapasso F, Yendamuri S, Matsuyama A, Alder H, Mori M, Croce CM:](http://www.ncbi.nlm.nih.gov/entrez/query.fcgi?db=pubmed&cmd=Retrieve&dopt=AbstractPlus&list_uids=12839965&query_hl=9&itool=pubmed_DocSum) **Allele loss and promoter hypermethylation of VHL, RAR-beta, RASSF1A, and FHIT tumor suppressor genes on chromosome 3p in esophageal squamous cell carcinoma.** *Cancer Res* 2003, **63**:3724-3728.
9. [Lee EJ, Lee BB, Kim JW, Shim YM, Hoseok I, Han J, Cho EY, Park J, Kim DH:](http://www.ncbi.nlm.nih.gov/entrez/query.fcgi?db=pubmed&cmd=Retrieve&dopt=AbstractPlus&list_uids=16564166&query_hl=9&itool=pubmed_DocSum) **Aberrant methylation of Fragile Histidine Triad gene is associated with poor prognosis in early stage esophageal squamous cell carcinoma.** *Eur J Cancer* 2006, **42**:972-980.
10. [Michalowski MB, de Fraipont F, Michelland S, Entz-Werle N, Grill J, Pasquier B, Favrot MC, Plantaz D:](http://www.ncbi.nlm.nih.gov/entrez/query.fcgi?db=pubmed&cmd=Retrieve&dopt=AbstractPlus&list_uids=16616114&query_hl=9&itool=pubmed_DocSum) **Methylation of RASSF1A and TRAIL pathway-related genes is frequent in childhood intracranial ependymomas and benign choroid plexus papilloma.** *Cancer Genet Cytogenet* 2006, **166**:74-81.
11. [Nakata S, Sugio K, Uramoto H, Oyama T, Hanagiri T, Morita M, Yasumoto K:](http://www.ncbi.nlm.nih.gov/entrez/query.fcgi?db=pubmed&cmd=Retrieve&dopt=AbstractPlus&list_uids=16598757&query_hl=9&itool=pubmed_DocSum) **The methylation status and protein expression of CDH1, p16(INK4A), and fragile histidine triad in nonsmall cell lung carcinoma: epigenetic silencing, clinical features, and prognostic significance.** *Cancer* 2006, **106**:2190-2199.
12. [Takada S, Morita K, Hayashi K, Matsushima T, Sawamura M, Murakami H, Nojima Y:](http://www.ncbi.nlm.nih.gov/entrez/query.fcgi?db=pubmed&cmd=Retrieve&dopt=AbstractPlus&list_uids=16313263&query_hl=9&itool=pubmed_DocSum) **Methylation status of fragile histidine triad (FHIT) gene and its clinical impact on prognosis of patients with multiple myeloma.** *Eur J Haematol* 2005, **75**:505-510.
13. [Tomizawa Y, Iijima H, Nomoto T, Iwasaki Y, Otani Y, Tsuchiya S, Saito R, Dobashi K, Nakajima T, Mori M:](http://www.ncbi.nlm.nih.gov/entrez/query.fcgi?db=pubmed&cmd=Retrieve&dopt=AbstractPlus&list_uids=15541815&query_hl=9&itool=pubmed_DocSum) **Clinicopathological significance of aberrant methylation of RARbeta2 at 3p24, RASSF1A at 3p21.3, and FHIT at 3p14.2 in patients with non-small cell lung cancer.** *Lung Cancer* 2004, **46**:305-312.
14. [Wali A, Srinivasan R, Shabnam MS, Majumdar S, Joshi K, Behera D:](http://www.ncbi.nlm.nih.gov/entrez/query.fcgi?db=pubmed&cmd=Retrieve&dopt=AbstractPlus&list_uids=16513840&query_hl=9&itool=pubmed_DocSum) **Loss of fragile histidine triad gene expression in advanced lung cancer is consequent to allelic loss at 3p14 locus and promoter methylation.** *Mol Cancer Res* 2006, **4**:93-99.
15. [Wu Q, Shi H, Suo Z, Nesland JM:](http://www.ncbi.nlm.nih.gov/entrez/query.fcgi?db=pubmed&cmd=Retrieve&dopt=AbstractPlus&list_uids=14660280&query_hl=9&itool=pubmed_DocSum) **5'-CpG island methylation of the FHIT gene is associated with reduced protein expression and higher clinical stage in cervical carcinomas.** *Ultrastruct Pathol* 2003, 27:417-422.
